# Supplementary material for: Research on the mechanism of promoting coordinated development of ecological well-being in rural counties through industrial transformation
Source: PLoS One. 2023 Sep 8;18(9):e0291232. doi: 10.1371/journal.pone.0291232 (PMC10490857; doi:10.1371/journal.pone.0291232)
Supplement: Attachment — Submitted filename: Response to Reviewers.docx [file pone.0291232.s002.docx]

**Response to Editor and Reviewers**

To Reviewer 1:

Thank you very much for your review and suggestions on the article; they have been greatly beneficial to the paper. We have made systematic revisions based on the changes you provided, and we have marked the modified areas in red. The specific changes are as follows:

**1.The abstract can be further refined. For example, the research results in the abstract can be summarized into three points.**

We have revised the abstract, taking your suggestions into account, condensing and categorizing the conclusion into 3 points, and restructuring the abstract to include the research objectives, research methods, research content, and main conclusions. The revised content can be found on the first page of the article. Once again, thank you for your suggestions for revision. They have been very helpful to us.

**2.The contributions of this paper require further clarification and improvement. It is suggested to further improve the innovation points.**

Thank you for your suggestions. We found that our previous manuscript might not have adequately described the core innovative aspects of this paper, so we have made corrections to the sections that describe the innovations. We have emphasized the innovation in the methods, perspectives, and content of the research, and highlighted the necessity and high value of this study. The specific modifications can be found on the page 2 of the article. Once again, thank you for your suggestions for revising the article.

**3.Some of the statements are written without reference; try to add reference with every statement in your paper.**

Thank you for your comments. Indeed, we discovered that some sentences did not have appropriate references to support them, so we have added citations where we felt they were needed in the article. The specific additions can be found on page 1, page 3, and page 4. If there are any areas where you still feel something is missing, we would be happy to make further additions.

**4. In the discussion part, the author should further explain the reasons for these results.**

Thank you for your suggestion. In the original manuscript, we only described the phenomenon without delving into its possible underlying reasons. Following your advice, we have revised this section of the manuscript. By considering both the phenomenon and its historical context, we conducted a deeper analysis of the essence of the phenomenon. The results of this analysis can be found on Page 17.

**5. The overall quality of English is good, but need to be checked carefully again. I suggest the authors should look for an English native speaker to further check the** **language of the paper.**

Thank you for affirming the level of English in our manuscript. We indeed spent a considerable amount of time polishing the language. We have once again sought out a scholar whose native language is English to make revisions to the manuscript's English. If there are still places that are not well-written, we would be very pleased to make further revisions.

**6. Some fresh paper can be used as ref. eg: Liu, F., Sim, J. Y., Sun, H., Edziah, B. K., Adom, P. K., & Song, S. (2023). Assessing the role of economic globalization on energy efficiency: Evidence from a global perspective. China Economic Review, 77, 101897. <https://doi.org/10.1016/j.chieco.2022.101897>.**

Thank you for your suggestion. We have updated the references in our manuscript. After consulting the literature you mentioned, we believe that it has a significant relationship with our research. The citation can be found on page 1. If you think there are other more suitable and recent references for this paper, we would be very pleased to make further updates.

**7.why nothing in the 3rd part of 2.3 Data sources?**

Yes, we made a mistake, and this section was erroneously deleted by us. We have corrected it and added back the complete information. Thank you for bringing this to our attention.

Thank you once again for taking the time to review and offer suggestions for our manuscript. Your valuable advice is crucial for us to enhance the quality of the article. We look forward to your next response. Please accept our most sincere blessings.

To Reviewer 2:

Thank you very much for your review and suggestions on the article; they have been greatly beneficial to the paper. We have made systematic revisions based on the changes you provided, and we have marked the modified areas in red. The specific changes are as follows:

**About Abstract:**

We have revised the abstract, taking your suggestions into account, condensing and categorizing the conclusion into 3 points, and restructuring the abstract to include the research objectives, research methods, research content, and main conclusions. The revised content can be found on the first page of the article. Once again, thank you for your suggestions for revision. They have been very helpful to us.

**About Introduction**

Thank you for your suggestions. We have identified many of the issues you pointed out in our introduction. Based on your recommendations for the manuscript, we have made the following modifications to the introduction:

(1) We have added some of the latest literature to make the logic of the article more coherent.

(2) We have rewritten the innovative points of the article, clarifying the objectives and advantages of the article, and emphasizing the importance of the research.

(3) We have revised the literature review to enrich this manuscript.

(4) We have made further language corrections to certain paragraphs, enhancing the readability of the article.

**About Variable Selection, Model Setting, and Research Methods Suggestions:**

Thank you for your suggestions. We have made certain revisions to this module:

(1) In addition to the original information about the data sources, we have included explanations for the accessibility of the data.

(2) We have provided detailed descriptions of the data processing methods and synthesis techniques.

(3) We have systematically explained the theoretical basis for the coupling coordination degree in the article.

(4) We have given a detailed overview of the application of the SDM model in the article.

**About Regional Overview Suggestions:**

Thank you for your suggestions. We have made certain revisions to this module:

We have provided a more detailed description and rewrite of the study area overview, taking into consideration not only the geographical features of the research area but also its economic conditions. We have emphasized the necessity of research in this region and the scarcity of county-level studies.

**About Research Methods Suggestions:**

Thank you for your suggestions. We have made certain revisions to this module:

(1) We have systematically described the spatiotemporal transformation trends of the coupling coordination degree.

(2) We have created a county-level transition matrix analysis diagram for the coupling coordination degree, exploring the transition status of the coordination.

(3) We have analyzed the reasons for the transition.

(4) We optimized the expression of the empirical conclusions and rewrote the impact mechanism for this module.

**About Discussion Suggestions:**

Thank you for your suggestion. In the original manuscript, we only described the phenomenon without delving into its possible underlying reasons. Following your advice, we have revised this section of the manuscript. By considering both the phenomenon and its historical context, we conducted a deeper analysis of the essence of the phenomenon. The results of this analysis can be found on Page 17.

**About Conclusions and Policy Recommendations Suggestions**

Thank you for your suggestions. We have made certain revisions to this module:

We have further revised the conclusion of the article, emphasizing the mechanism of impact, and we have also made corrections to the policy recommendations. Based on the model regression results, we have provided more appropriate policy suggestions. We have proposed policy recommendations to drive regional development through regional development and have provided suitable data support to ensure that the recommendations are trustworthy.

Thank you once again for taking the time to review and offer suggestions for our manuscript. Your valuable advice is crucial for us to enhance the quality of the article. We look forward to your next response. Please accept our most sincere blessings.
